# The four-transmembrane protein MAL2 and tumor protein D52 (TPD52) are highly expressed in colorectal cancer and correlated with poor prognosis

**DOI:** 10.1371/journal.pone.0178515

**Published:** 2017-05-31

**Authors:** Jingwen Li, Yongmin Li, He Liu, Yanlong Liu, Binbin Cui

**Affiliations:** Department of Colorectal Surgery, Harbin Medical University Cancer Hospital, Harbin, China; University of South Alabama Mitchell Cancer Institute, UNITED STATES

## Abstract

The four-transmembrane protein MAL2 and tumor protein D52 (TPD52) have been shown to be involved in tumorigenesis of various cancers. However, their roles in colorectal cancer (CRC) remain unclear. In this study, we explored the expressions of MAL2 and TPD52 in tumor specimens resected from 123 CRC patients and the prognostic values of the two proteins in CRC. Immunohistochemical analyses showed that MAL2 (P<0.001) and TPD52 (P<0.001) were significantly highly expressed in primary carcinoma tissues compared with adjacent non-cancerous mucosa tissues. And TPD52 exhibited frequent overexpression in liver metastasis tissues relative to primary carcinoma tissues (P = 0.042), while MAL2 in lymphnode and liver metastasis tissues showed no significant elevation. Real-time quantitative PCR (RT-qPCR) showed the identical results. Correlation analyses by Pearson’s chi-square test demonstrated that MAL2 in tumors was positively correlated with tumor status (pathological assessment of regional lymph nodes (pN, P = 0.024)), and clinic stage (P = 0.017). Additionally, the expression of TPD52 was detected under the same condition and was shown to be positively correlated withtumor status (pathological assessment of the primary tumor (pT, P = 0.035), distant metastasis (pM, P = 0.001)) and CRC clinicopathology(P = 0.024). Kaplan-Meier survival curves indicated that positive MAL2 (P<0.001) and TPD52 (P<0.001) expressions were associated with poor overall survival (OS) in CRC patients. Multivariate analysis showed that MAL2 and TPD52 expression was an independent prognostic factor for reduced OS of CRC patients. Moreover, overexpression of TPD52 in CRC SW480 cells showed an increased cell migration (P = 0.023) and invasion (P = 0.012) through inducing occurrence of epithelial-mesenchymal transition (EMT) and activating focal adhesion kinase (FAK)-mediated integrin signalling and PI3K⁄Akt signalling.Whereas TPD52-depleted cells showed the reverse effect. These data suggested that MAL2 and TPD52 might be potential biomarkers for clinical prognosis and might be a promising therapeutic target for CRC.

## Introduction

Colorectal cancer (CRC) is known as a widespread malignant tumor, representing the third most common cancer in both men and women [1]. It is a pity that only a small fraction of patients with metastatic CRC can undergo curative resection and disease-free survival [2]. The lack of reliable biomarkers lead to late diagnosis of malignancy, distant metastasis and consequently low 5-year survival rates. Thus, identifying prognostic markers might help to reduce CRC mortality [3,4] and incidence [5,6]. To clarify the causes of frequent therapy failure, the underlying molecular basis of the histologically heterogeneous group of tumors needs to be understood. A plenty of cytogenetic and comparative expression studies were performed. The four-transmembrane protein MAL2 whose gene is located on chromosome 8q, commonly identified to be correlated with membrane apposition events[7], was found to be overexpressed or increased in copy number in some human cancers [8–17]. MAL2 expression has been shown to be elevated by independent expression microarray studies in ovarian cancer [18–20] and increased at RNA and protein levels in renal cell [17,21] and breast cancers[11]. However, the expression of MAL2 in colorectal cancer has not been still investigated, and its potential clinical significance remains unclear.

Tumor protein D52 (TPD52), a member of the similarly-named gene and protein family [22], is the product of another gene on chromosome 8q, which is known to bind MAL2 [23]. The expression of TPD52 in normal secretory epithelia has been demonstrated to regulate exocytotic secretion [24]. However, TPD52 gene showed overexpression and copy number increases in multiple human cancers [25] and its exogenous expression was associated with the increased anchorage-independent growth and cell proliferation [11,26,27], and metastasis in vivo in immunocompetent hosts [26]. It was indicated that TPD52 expression in ovarian cancer tissue was higher compared with normal ovarian epithelium [28], and TPD52 was found to be overexpressed in prostate cancer [29,30] and breast cancer [31,32]. Additionally, TPD52 overexpression in colorectal tissue samples was identified by MS analysis compared to normal tissue [33]. However, the clinical significance of TPD52 overexpression in colorectal cancer has not been investigated. Moreover, TPD52 was demonstrated to promote tumorigenesis and metastasis of NIH3T3 fibroblasts [34] and TPD52 exogenous overexpression enhanced cellular migration of prostate cancer [35]. TPD52 overexpression was found in lung squamous cell carcinoma (SCC), and TPD52 silencing inhibited migration and invation in lung SCC cells [36]. However, whether TPD52 regulates cellular motility in colorectal cancer is still unknown.

In the present study, we investigated the expression of MAL2 and TPD52 in colorectal cancer clinical specimens and the potential clinical significance. Further, the role of TPD52 expression in cell migration and invation of colorectal cancer and underlying molecular mechanism was established.

## Materials and methods

### Patient and tissue specimens

The formalin-fixed paraffin-embedded tissue samples used to IHC were collected from 123 CRC patients undergoing surgery in 2005–2010, and were grouped as tumor tissue adjacent non-cancerous mucosa, primary carcinoma, lymphnode metastasis and liver metastasis tissue. And all of the the mucosa were collected from the 123 CRC patients. Similarly, there were matching lymphnode metastasis tissues for 58 primary carcinomas and matching liver metastasis tissue for 38 primary carcinomas. Primary carcinomas were assessed according to the 7th edition American Joint Committee on Cancer (AJCC) staging system. All data and tissues of CRC patients as paraffin blocks were collected from Harbin Medical University Cancer Hospital. This study comprised 73 men and 50 women aged from 29 to 88 years old, mean age was 60 years. Of those, in accordance with the postoperative pathological, the 123 patients were divided into four periods based on the International Union against Cancer 2002 TNM staging system and WHO classification criteria [37]. The follow-up period was0.6–106.8 months.The research into the standard set is as follows: (1) All the patients with primary tumor have been completely resectioned by surgery; (2) No patients received neoadjuvant therapy before surgery. The study was approved by the Ethics Committee of Harbin Medical University Cancer Hospital, Harbin, China. And all of the donors or the next of kins had written the informed consent for use of the tissue sample in research.

### Pathology review

Hematoxylin and eosin-stained slides were reviewed by two surgical pathologists with CRC professional knowledge, according to WHO and NCCN guideline the slides were graded and subtyped.

### Immunohistochemistry (IHC)

The normal mucosa and primary carcinoma tissues used for IHC were obtained from the same patient. The formalin-fixed, paraffin-embedded sections (4μm) were deparaffinized, rehydrated, and then quenched with 3% H_2_O_2_ for 10 min. Antigen retrieval were performed by autoclave in 10 mM sodium citrate buffer (pH6.0) for 3 min at 121°C. The sections were then immersed in a 0.3% hydrogen peroxide solution for 30 min to block endogenous peroxidase activity. After washing with PBS for 5 min, the sections were incubated with rabbit polyclonal MAL2 antibody (#ab75347, 1:200dilution, Abcam, Shanghai, China) and TPD52 antibody (#HPA027916, 1:200 dilution; Human Protein Atlas; Atlas Antibodies, Stockholm, Sweden) overnight at 4°C. A negative control was performed by replacing the primary antibody with a normal rabbit IgG antibody, indicating the specifity of antibody. Then the sections were incubated with Horseradish peroxidase (HRP)-conjugated mouse anti-rabbit secondary antibody (PV6001, 1:1000 dilution, Zhongshan Goldbridge Biotechnology, China) for 20 min. 3, 3′-diaminobenzidine tetrahydrochloride (DAB) was used for development and the slides were counterstained using Mayer’s hematoxylin.

Two experienced pathologists blinded to the clinicopathological information scored the MAL2 and TPD52 levels in tumor cells by assessing (a) the proportion of positively stained cells: (0, <5%; 1, 6 to 25%; 2, 26 to 50%; 3, 51 to 75%; 4, >75%) and (b) the signal intensity: (0, no signal; 1, weak; 2, moderate; 3, strong). The score was determined by multiplying a and b [38]. The levels of the two proteins were obtained by counting the positively and negatively stained cells in five to ten separate 400× high-power microscopic fields and calculating the mean percentage of positively stained cells among the total cells per field.

### Real-time quantitative PCR (RT-qPCR)

Total RNA in cells was extracted using RNA isolation kit (A&A Biotechnology, Poland). cDNA was obtained by reverse transcription PCR (RT-PCR)using RevertAid™ First Strand cDNA synthesis kit (Fermentas International, Lithuania) and was amplified using TaqMan® Gene Expression Assay (Applied Biosystems), and the specific primers for target proteins were used. mRNA expression of target proteins were calculated using the formula 2^ΔΔCt^ and was normalized to the level of GAPDH. The value of mRNA in tumor cells was demonstrated as the relative value of mRNA in control cells.

### Cell line and culture

The human CRC cell line SW480 was obtained from the Cancer Research Institute of Harbin Medical University. The cell lines were maintained in RPMI-1640 (Gibco-BRL, Carlsbad, CA, USA) supplemented with 10% fetal bovine serum (ExCell Bio, Shanghai, China), 100 μg/ml streptomycin and 100 units/ml penicillin (Invitrogen, Carlsbad, CA, USA) and cultured at 37°C in a humidified atmosphere containing 5% CO2.

### Overexpression and RNA interference of TPD52

The PG307-TPD52 plasmid was constructed by subcloning TPD52 fragment (GenBank NM_005079.3)between SalI and BamHI restriction sites, representing the 91-bp 5′UTR, 555-bp coding sequence and 2599-bp 3′UTR, into the PG307 expression vector [39]. The pcDNA of TPD52 clone was confirmed by digesting minipreps with SalI and BamHI, and by DNA sequencing (BGI, Shanghai, China). The PG307-TPD52 overexpression vector or empty PG307 vector as negative control were stably transfected into cells, as described previously [39].

siTPD52 (P/N: HSS120730 and HSS120731, Invitrogen, Carlsbad, CA, USA), and negative control siRNA (P/N: 4390843, Invitrogen) were used in this study. SW480 cells in Opti-MEM medium (cat.no. 31985070; Thermo Fisher Scientific, Waltham, MA, USA) were transfected with Lipofectamine RNAiMAX transfection reagent (P/N: 56532, Invitrogen) with 10 nM siRNA according to the instruction.

### Western blot analysis

Cells were washed twice with PBS, and were collectedand lysed in lysis buffer (50mM Tris-HCl (pH 7.4), 1mM EDTA, 1% NP40, 150mM NaCl, 10mM NaF, 1mM Na_3_VO_4_) containing a protease inhibitor cocktail (Roche, NJ, USA). Proteins were extracted from lysed cells. 20 μg Protein lysates were separated by 10% SDS-PAGE (Bio-Rad Laboratories, Inc., Hercules, CA, USA) and transferred to Immobilon-P membranes (Millipore, MA, USA). Immunoblotting was performed using diluted rabbit polyclonal anti-TPD52 (#HPA027916, 1:250 dilution; Human Protein Atlas; Atlas Antibodies, Stockholm, Sweden), rabbitpolyclonal anti-(human Akt) (#9272, 1:1000 dilution; Cell Signalling Technology, Frankfurt,Germany) or rabbit polyclonal anti-phospho-Aktat Ser473 (#9271, 1:1000 dilution; Cell Signalling Technology), mouse monoclonal anti-E-cadherin (sc-21791, 1:2000, Santa Cruz, CA, USA), mouse monoclonalanti-Vimentin (sc-6260, 1:2000, Santa Cruz), rabbit polyclonal anti-FAK antibody (#3285, 1:1000, Cell Signaling Technology, Beverly, MA, USA) and mouse monoclonal anti-GAPDH antibodies (#MAB374, 1:10,000 dilution; Chemicon International, Inc., Temecula, CA, USA) overnight at 4°C, and HRP-conjugated secondary antibodies: goat anti-mouse (#sc-2005, 1:2000 dilution; Santa Cruz, CA, USA) and goat anti-rabbit IgG (#sc-2004, 1:2000 dilution; Santa Cruz, CA, USA) were used to incubation for 1 h at room temperature. ECL-detecting reagent (Amersham Biosciences, Buckinghamshire, England) was used to development.

### Transwell assay

Cell migration were evaluated by Transwell assay in 24-well transwell plates (8 μm pore size, Corning Costar, USA) [40]. RPMI-1640 containing 20% fetal bovine serum in the lower chamber served as the chemoattractant. Cells (5×10^5^/well) were incubated at 37°C for 24 h. The cells on the undersurface of the upper chamber were presented by crystal violet (Amresco, USA) staining for 10 minutes at room temperature, and then were observed using a light microscope (Olympus, Japan) and chose six random fields at 100× magnification to score the average cell coverage. Invasion assay was performed as the same procedure as migration assay. Differently, the chamber inserts were coated with 1 mg/mL Matrigel membrane (BD Biosciences, USA) and the invasive cells were detected after incubation for 48 h.

### Statistical analysis

The IBM SPSS Statistics version 21.0 (IBM Co., Armonk, NY, USA) software was used for the statistical analysis. A chi-square test was used to analyze the relationship between the expression of MAL2 or TPD52 and various clinicopathologic parameters. Overall survival were evaluated to determine the prognostic value of the two proteins expression using the Kaplan-Meier method. P<0.05 was considered statistically significant. Data was presented as the mean ± standard error of the mean (SEM).

## Results

### MAL2 and TPD52 are overexpressed in CRC

**Fig 1:**
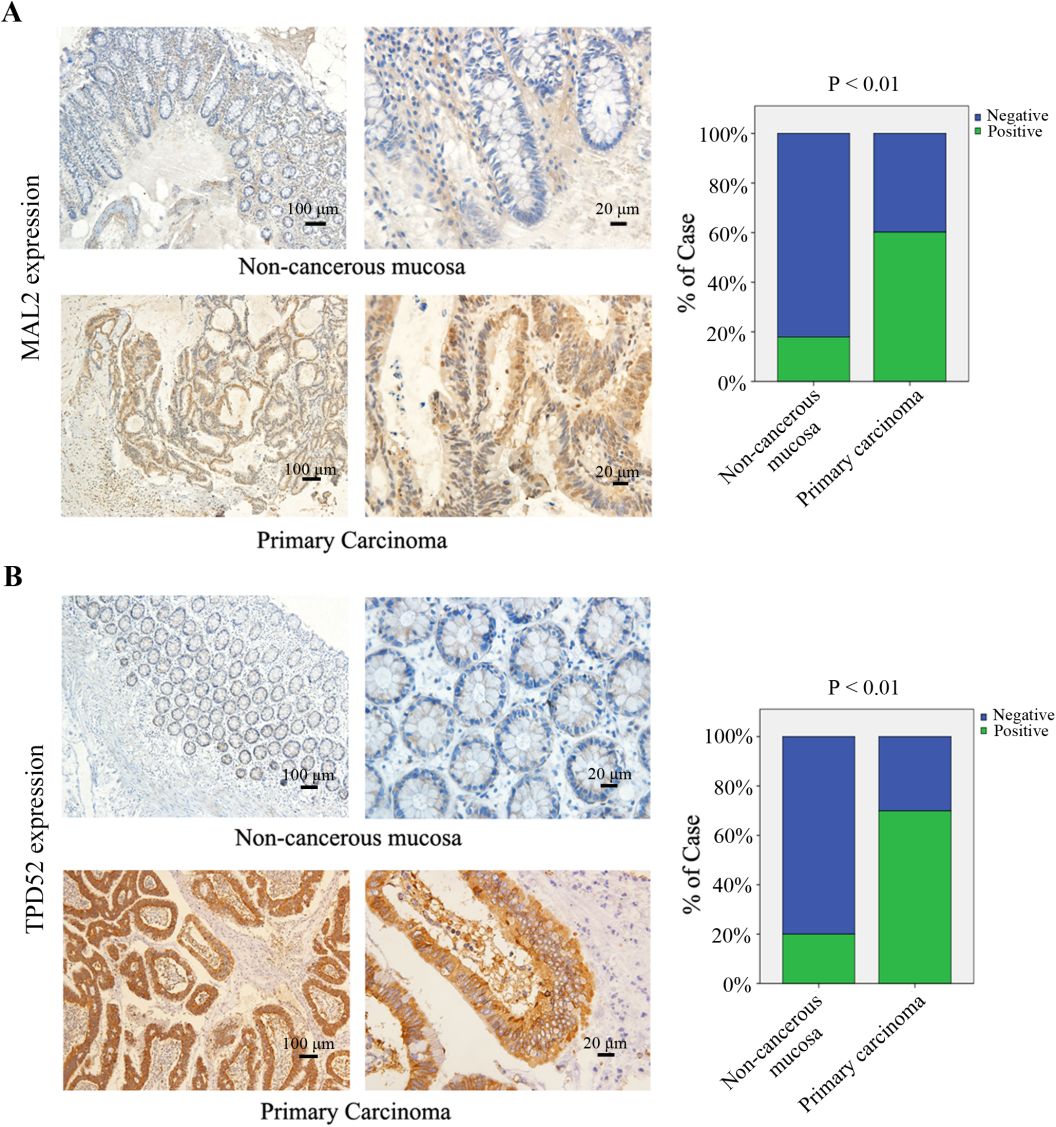
Immunohistochemical staining for MAL2 and TPD52 in non-cancerous mucosa and primary carcinoma tissues of patients with CRC. (A,B) Expression levels of MAL2 and TPD52 in non-cancerous mucosa and primary carcinoma tissues from patients with CRC were detected by immunohistochemical staining (×100, 100 μm; ×400, 20 μm). MAL2 and TPD52 expression levels were quantified as shown as the right bar charts. **P<0.01 compared with non-cancerous mucosa.

**Fig 2:**
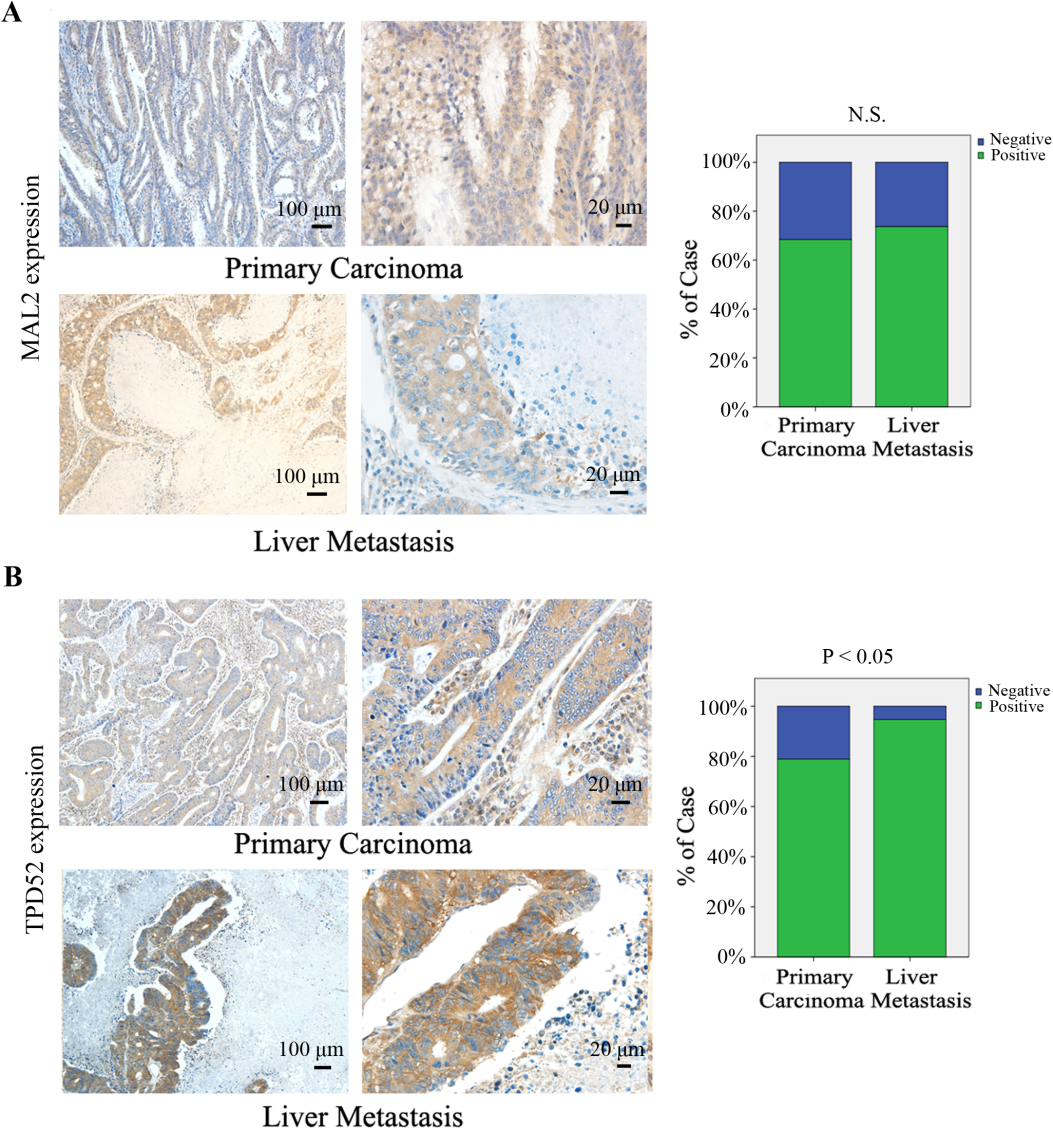
Immunohistochemical staining for MAL2 and TPD52 in human CRC tissues. (A,B) Expression levels of MAL2 and TPD52 in primary carcinoma tissuesand liver metastasis tissues from patients with CRC were detected by immunohistochemical staining (×100, 100 μm; ×400, 20 μm). MAL2 and TPD52 expression levels were quantified as shown as the right bar charts. *P<0.05 compared with primary carcinoma; N.S., no significance.

**Fig 3:**
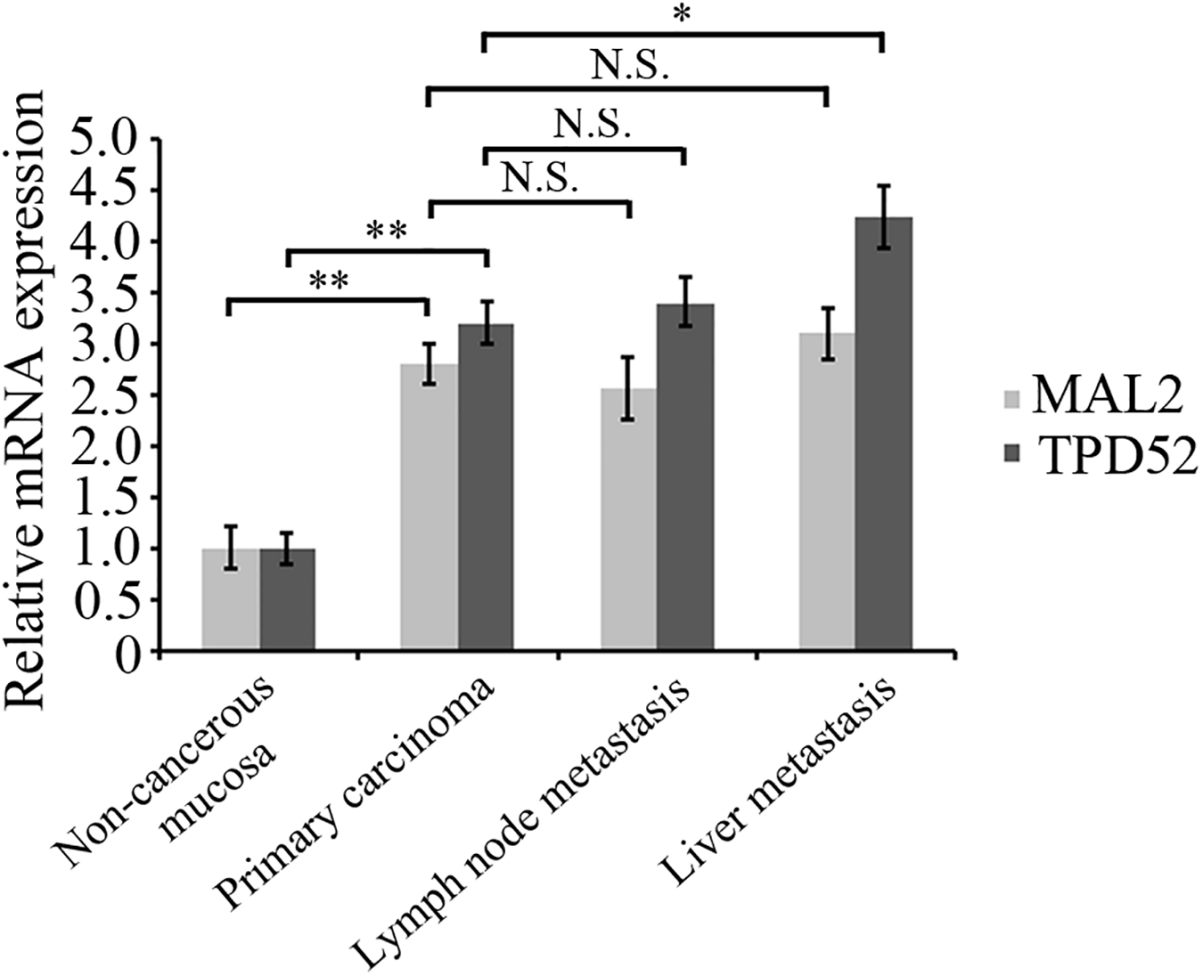
mRNA expression of MAL2 and TPD52 in human CRC. RT-qPCR was used to detect the mRNA expression of MAL2 and TPD52 in patient tissues. GAPDH was detected as the internal reference. The bar graph showed the amount of MAL2 and TPD52 protein normalized to the amount of GAPDH. *P<0.05, **P <0.01; N.S., no significance.

To validate whether MAL2 and TPD2 are involved in CRC carcinogenesis, we examined expression of the two proteins in the paired tumors, lymph nodes, liver metastasis and adjacent non-cancerous mucosa tissues from CRC patients (n = 123) by immunohistochemical staining.As shown in Table 1, both MAL2 (n = 123, P<0.001) and TPD2 (n = 123, P<0.001) expressions were significantly increased in CRC primary carcinoma tissues compared with that in adjacent non-cancerous mucosa (Fig 1A and 1B). Similarly, the TPD2 expression in liver metastasis tissues (n = 38, P = 0.042) remarkably increased relative to that in primary carcinoma tissues (Fig 2B). However, MAL2 (n = 58, P = 0.55) and TPD52 (n = 58, P = 0.672) protein expressions in lymphnode metastasis tissues (data not shown) and MAL2 expression in liver metastasis tissues (n = 38, P = 0.782; Fig 2A) did not exhibit significant elevation relative to primary carcinoma groups. RT-qPCR was used to detect the mRNA expression of MAL2 and TPD52 in patient tissues and demonstrated that the indentical results to immunohistochemical staining (Fig 3). These results suggested that MAL2 and TPD52 were involved in tumorigenesis and TPD52 might be associated with liver metastasis of CRC.

**Table 1:** Expression comparison for MAL2 and TPD52 in tissues of patient with CRC.

Variable	No.	MAL2				TPD52			
		Negative (%)	Positive (%)	χ2	p -value	Negative (%)	Positive (%)	χ2	p -value
1				46.19	<0.001*			61.09	<0.001*
Non-carcerous	123	101(82.1)	22(17.9)			98(80.0)	25(20.0)		
Primary carcinoma	123	49(39.8)	74(60.2)			37(30.1)	86(69.1)		
2				0.357	0.55			0.18	0.672
Primary carcinoma	58	17(29.3)	41(70.7)			14(24.1)	44(75.9)		
Lymph node	58	20(34.5)	38(65.5)			16(27.6)	42(72.4)		
3				0.076	0.782			4.145	0.042*
Primary carcinoma	38	12(31.6)	26(68.4)			8(21.1)	30(78.9)		
Liver metastasis	38	10(26.3)	28(73.7)			2(5.3)	36(94.7)		

*P < 0.05, statistically significant, Pearson’s X^2^ test.

### MAL2 and TPD52 correlate with clinicopathologic features

The correlations between MAL2 and TPD52 expression and clinicopathologic features in tissue samples from 123 CRC patients were investigated as demonstrated in Table 2. The patients were divided into four groups (a MAL2-positive group, a MAL2-negative group, a TPD52-positive group and a TPD52-negative group) based on the medians of immunohistochemical variable values in diverse cell subsets. MAL2 was shown to be positively correlated with tumor status (pathological assessment of regional lymph nodes (pN, P = 0.024)) and clinic stage (P = 0.017). However, there were no significant correlation between MAL2 expression and primary carcinoma location (P = 0.065), tumor status (pathological assessment of the primary tumour (pT, P = 0.191); pathological assessment of metastasis (pM, P = 0.108)). TPD52 expression in tumors was shown to be positively correlated with tumor status (pathological assessment of the primary tumour (pT, P = 0.035); metastasis (pM, P = 0.001)), and clinic stage (P = 0.024); Whereas, TPD52 expression was not associated with pathological assessment of regional lymph nodes (pN, P = 0.175) and tumor size (P = 0.708) of CRC patients. In addition, the tumor marker CEA was also shown to be positively associated with MAL2 positive expression (P = 0.005), suggesting that MAL2 expression in tumor cells was a potential predictive factor for CRC.

**Table 2:** MAL2 and TPD52 staining in tumor cells and associations with clinicopathologic characteristics.

Variable	No.	MAL2				TPD52			
		Negative (%)	Positive (%)	X^2^	p -value	Negative (%)	Positive (%)	X^2^	p -value
**Age(years)**				0.109	0.741			0.046	0.829
<60	68	25 (36.8)	40 (63.2)			21 (30.9)	47 (69.1)		
≥60	55	24 (43.6)	34 (56.4)			16 (32.0)	39 (68.0)		
**Gender**				3.219	0.073			3.272	0.07
Male	73	24 (32.9)	49 (67.1)			18 (24.7)	55 (75.3)		
Female	50	25 (50.0)	25 (50.0)			19 (38.0)	31 (62.0)		
**Location**				3.402	0.065			1.481	0.224
Colon	50	15 (30.0)	35 (70.0)			12 (24.0)	38 (76.0)		
Rectum	73	34 (46.6)	39 (53.4)			25 (34.2)	48 (65.8)		
**Tumor size(cm)**				0.001	0.971			0.14	0.708
<5	59	23 (39.0)	36 (61.0)			19 (32.2)	40 (67.8)		
≥5	64	26 (40.6)	38 (59.4)			18 (28.1)	46 (71.9)		
**Grade**				0.002	0.966			0.14	0.708
Low	51	21 (41.2)	30 (58.8)			13 (25.5)	38 (74.5)		
High	72	30 (41.7)	42 (58.3)			24 (33.3)	48 (66.7)		
**pT classification**^a^				1.714	0.191			4.463	0.035*
T1-T3	49	23 (46.9)	26 (53.1)			20 (40.8)	29 (59.2)		
T4	74	26 (35.1)	48 (64.9)			17 (23.0)	57 (77.0)		
**pN classification**^b^				5.075	0.024*			1.843	0.175
N0	65	32 (49.2)	33 (50.8)			23 (35.4)	42 (64.6)		
N1-N2	58	17 (29.3)	41 (70.7)			14 (24.1)	44 (75.9)		
**pM classification**^c^				2.579	0.108			11.71	0.001*
M0	85	39 (45.9)	46 (54.1)			34 (40.0)	51 (60.0)		
M1	38	10 (26.3)	28 (73.7)			3 (7.9)	35 (92.1)		
**AJCC stage**^d^				10.15	0.017*			9.425	0.024*
I	25	16 (64.0)	9 (36.0)			14 (56.0)	11 (44.0)		
II	29	13 (44.8)	16 (55.2)			11 (37.9)	18 (62.1)		
III	31	11 (35.5)	20 (64.5)			8 (25.8)	23 (74.2)		
IV	38	9 (23.7)	29 (76.3)			4 (10.5)	34 (89.5)		
**CEA**				8.044	0.005*			1.086	0.297
<5 (Negative)	61	32 (52.5)	29 (47.5)			21 (34.4)	40 (65.6)		
≥5 (Positive)	62	17 (27.4)	45 (72.6)			16 (25.8)	46 (74.2)		
**CA199**				0.936	0.333			0.156	0.693
<37 (Negative)	97	42 (43.3)	55 (56.7)			29 (29.9)	68 (70.1)		
≥37 (Positive)	26	7 (26.9)	19 (73.1)			8 (30.8)	18 (69.2)		
**Total**	123	49 (39.8)	74 (60.2)			37 (30.1)	86 (69.9)		

*P < 0.05, statistically significant, Pearson’s X^2^ test.Basis age cutoff was 60 years, tumor size cutoff was 5 cm, CEA cutoff was 5, and CA199 cutoff was 37.

^a^pathological assessment of the primary tumour

^b^pathological assessment of the regional lymph nodes

^c^pathological assessment of the distant metastasis

^d^American Joint Committee on Cancer.

### Correlation of MAL2 and TPD52 expression with prognosis in CRC

**Fig 4:**
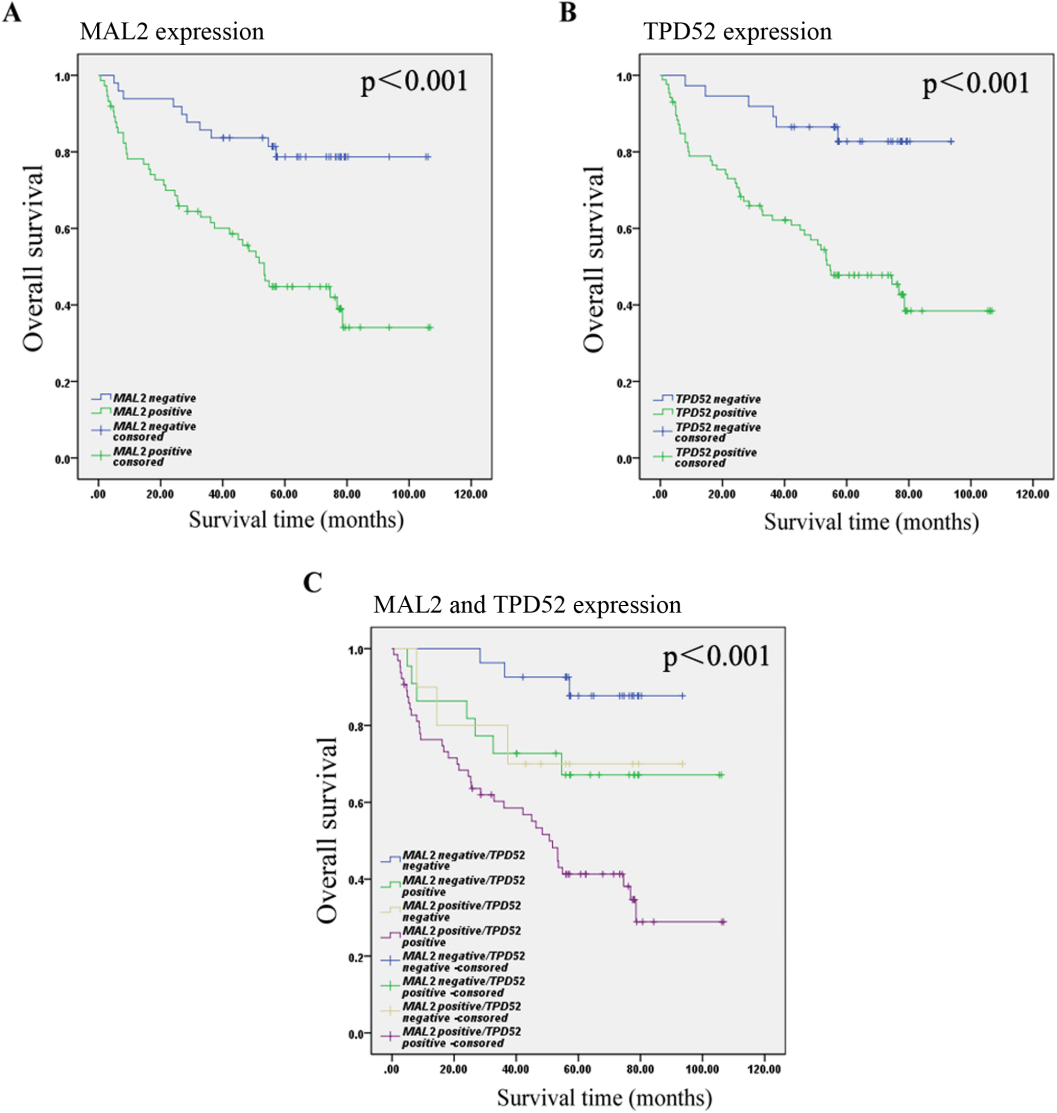
Kaplan-Meier survival analysis in patients with CRC. (A) Overall survival curves for patients according to the negative (n = 49) and positive (n = 74) expression levels of MAL2 of immunohistochemical variables in tumor cells. (B) Overall survival curves for patients according to the negative (n = 37) and positive (n = 86) expression levels of TPD25 of immunohistochemical variables in tumor cells. (C) Overall survival curves for patients according to the positive and negative expression of MAL2 and TPD52 of immunohistochemical variables in tumor cells.

The Kaplan-Meier curves with a log-rank test demonstrated that patients with positive MAL2 or TPD52 expression seemed to be remarkably correlated with poor OS relative to those CRC patients with negative MAL2 or TPD52 expression (P<0.001; P<0.001; P<0.001; Fig 4A–4C). Among the 49 patients with negative MAL2, the median survival time was 89.709±4.714 months; among the 74 patients with positive MAL2, the median survival time was 57.920±5.032 months (p<0.001). Among the 37 patients with negative TPD52, the median survival time was 82.947±4.065 months; among the 86 patients with positive TPD52, the median survival time was 60.842±4.708months (p<0.001). The median survival time of the patients with negative MAL2/negative TPD52, negative MAL2/positive TPD52, positive MAL2/negative TPD52, or positive MAL2/positive TPD52 was 87.289±3.459, 78.965±8.618, 71.500±10.895, and 54.596±5.313 months (p<0.001), respectively. These results suggested that the MAL2 and TPD52 expressions in tumor cells were the promising predictive factor for OS of CRC patients. Furthermore, we determined that the expression of MAL2 and TPD52 in tumor cells was an independent predictor of OS according to the multivariate Cox model analysis (Table 3).

**Table 3:** Multivariate cox analyses for OS of 123 patients with CRC.

Variable	HR (95%CI)	p-value
AJCC stage (I-II/III-IV)	1.884 (1.196–3.300)	<0.001*
T stage (T1-3/T4)	1.743 (1.056–3.158)	0.002*
MAL2 in Tumor cells (Negative/Posive)	1.914 (1.092–3.515)	<0.001*
TPD52 in Tumor cells (Negative/Posive)	1.845 (1.033–3.158)	<0.001*
Combination of MAL2 and TPD52 in Tumor cells (Negative/Posive)	1.273 (1.088–2.115)	<0.001*

*P < 0.05, statistically significant.

### TPD52 regulates migration and invasion in CRC SW480 cells

**Fig 5:**
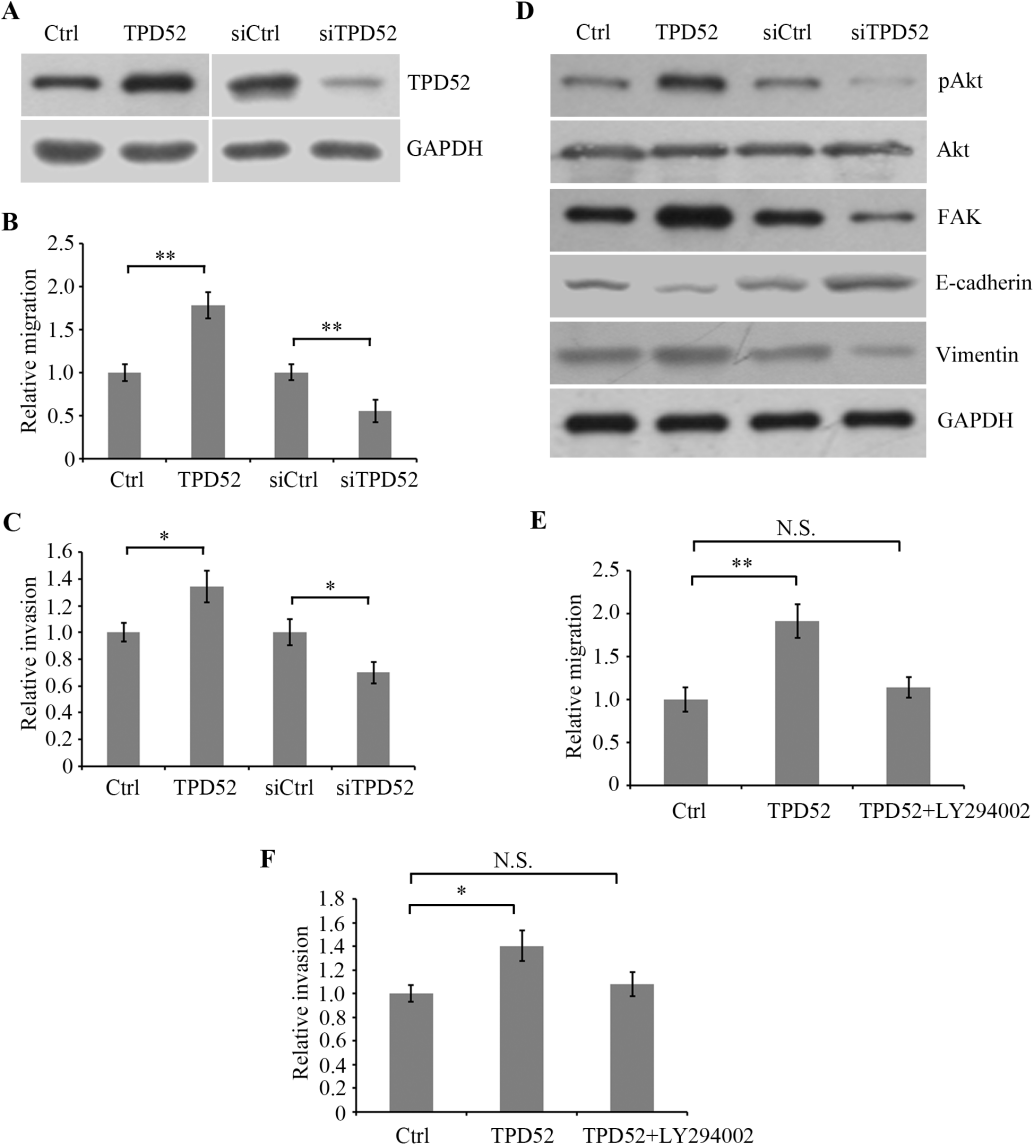
TPD52 regulates migration and invasion in CRC SW480 cells. (A) SW480 cells were transfected with PG307-TPD52 overexpression vector (TPD52) or empty PG307 vector as negative control (Ctrl). Also, SW480 cells were transfected with siRNA for TPD52 (siTPD52) or control siRNA (siCtrl). Western blot analysis was used to detect TPD52 protein expression in cells using TPD52 antibody. GAPDH was detected as internal reference. (B,C) The cellular migration and invasion abilities were evaluated by Transwell assay. (D) Akt (Ser473) phosphorylation was detected by Western blotting using the corresponding antibody and Akt protein was examined. (E,F) TPD52-overexpressed SW480 cells were treated with 2 μM LY294002, the PI3K specific inhibitor, for 24 h, and migration and invasion abilities of TPD52-overexpressed and control cells were evaluated. *P<0.05, **P<0.01 compared with control cells; N.S., no significance.

To verify the role of TPD52 in the metastasis of CRC, we overexpressed or knocked down TPD52 in CRC SW480 cells and examined the migration and invasion ability. Western blotting indicated that TPD52 protein was significantly increased in TPD52-overexpressed cells compared with empty vector transfected control cellsand decreased in TPD52-silenced cells compared with non-silenced control cells (Fig 5A). Transwell assay showed that TPD52 overexpression significantly promoted migration andinvasion of SW480 cells, while TPD52 silencing showed the adverse effect (Fig 5B and 5C). These data confirmed that TPD52 played an important role in CRC metastasis and was positively associated with celluar motility in SW480 cells.It has been shown that the *PrLZ* gene, encoding TPD52 or a member of TPD52 family, is reactivated and its expression is positively associated with cancer progression [41] and is able to activate PI3K/Akt signalling to regulate progression and development in prostate cancer [42]. We found that TPD52 overexpression activated PI3K⁄Akt pathway by stimulatig Akt (Ser473) phosphorylation, whereas TPD52 silencing attenuated the phosphorylation (Fig 5D). In addition, we showed that TPD52 overexpression induced EMT characterized by downregulation of E-cadherin and the upregulation of Vimentin [43], and the expression of FAK, a member of integrin signaling pathway [44]. And TPD52 silencing played an adverse role. Further, the PI3K specific inhibitor LY294002 was used to block PI3K/Akt signal pathway. It was shown that LY294002 markedly inhibited TPD52 overexpression-induced migration and invasion (Fig 5E and 5F), suggesting that PI3K/Akt signal pathway was involved in TPD52-mediated cellular migration and invasion, which might be associated with metastasis of CRC.

## Discussion

The prevalence of colorectal cancer (CRC), one of the most common cancers. Various of biomarkers that correlate with clinical significances have been explored in scientific research or used in clinical practice, however, with its limited diagnose application. Therefore, identifying prognostic markers might be significant for reducing CRC mortality and thus improving treatment. MAL2 was shown to be overexpressed in a variety of human cancers [8–17]. In the present study, we found that MAL2 was significantly highly expressed in primary carcinoma tissues relative to adjacent mucosa tissues from CRC patients, implying that MAL2 was associated with tumorigenesis of CRC. Additionally, MAL2 in tumors was demonstrated to be positively correlated with tumor status (pathological assessment of the regional lymph nodes), and clinic stage. And we found that positive MAL2 expression was associated with poor OS in CRC patients. The frequent overexpression of MAL2 in CRC primary tumor tissues and its low expression in non-cancerouslesions, implieda potential application for MAL2 in tumor monitoring and detection, particularly in high-grade carcinoma, which was consistent with the previous study of MAL2 in breast and ovarian cancer[11,18–20]. It has been shown that highly metastatic cells demonstrates the overexpression of MAL2 and high expression of MAL2 is associated with a lesser survival of postoperative patients and a high rate of distant metastasis in pancreatic cancer [45]. However, MAL2 expression in lymph node and liver metastasis tissues showed no significant elevation compared with primary carcinoma in our study, suggesting that MAL2 might not be directly correlated with tumor metastasis through lymph node and liver. Moreover, RT-qPCR showed the identical results to immunohistochemical analyses, indicating that RNA expression of MAL2 or gene copy number in tumor tissues was increased. The overexpression of MAL2 was reported to be associated with increased gene copy in various cancers. It has been shown that MAL2 is co-expressed with EpCAM [46], and MAL2 binds the oncoprotein MUC1 [47]. However, how MAL2 overexpression regulates tumorigenesis in CRC is still unclear, which might be involving the modulation on cancer-related signal pathways or cell-cell interactions [7]. Interesting, we found that there was a broad cytoplasmic expression of MAL2 in carcinoma tissues of CRC, while rare in non-cancerous tissues, which was also reported in breast cancer [11] and renal cell cancer [17], suggesting that the oncogenic function of MAL2 was carried out in the cytoplasm.

A plenty of studies showed that TPD52 was overexpressed in some human cancers and in association with carcinoma metastasis [29–36]. The overexpression of TPD52 was shown in primary carcinoma tissues, lymph node and liver metastasis tissues relative to non-cancerous mucosa tissues, suggesting the correlation between TPD52 and tumorigenesis and metastasis of CRC. TPD52 was exogenously overexpressed or knocked down, showing enhanced or inhibited migration and invasion in CRC SW480 cells. Several molecular events were involved in transformation of normal to tumor cells, even to metastasis [48]. It was reported that PI3K/Akt signal patyway was activated by PrLZ [42], sharing homology with TPD52. Hence, it was speculated that TPD family proteins might stimulate Akt signalling. In our study, TPD52 overexpression increased the phosphorylation of Akt at Ser473 and PI3K inhibitor LY294002 blocked the induction of TPD52 on cellular migration and invasion. The data indicated the involvement of PI3K/Akt signalling in TPD52-induced cellular motility. TPD52 expression in NIH3T3 cells promoted metastasis by inducing the expression of several genes [19]. It has been shown that integrins plays an important role in the formation of metastatic cells [49,50] and integrins avβ3 mediates cell migration of LNCaP cells to vitronectin and activates PI3K/Akt pathway by phosphorylating Akt at Ser473 [51]. Akt is activated via the phosphorylation mediated by PI3 kinase, and thus phosphorylates some substrates to regulate biological processes, including cell proliferation, survival and migration [42]. We showed that TPD52 expression activates the PI3K/Akt pathway. Whether the activation of the avβ3 signalling is involved in TPD52 induced cell migration via PI3K/Akt pathway remains to be established.Moreover, we found that FAK was regulated by TPD52 expression in cells. FAK plays an important role in integrin signaling [43,52–54]. It can be activated by integrin signaling and regulate a number of signal pathways, including STAT1, Ras-MAPK, and PI3K signaling [43,55,56], and thus stimulates cell proliferation and transformation. Integrins can mediate cell adherin and signal transduction between cell and ECM [57] and is an important regulater in cell proliferation, apoptosis, migration, and metastasis [58,59]. Therefore, it was possible that TPD52 modulated cell migration and invasion by inducing EMT and FAK-mediated integrin signalingand thus activating PI3K signaling. Additionally, like MAL2, TPD52 mRNA level was detected by RT-qPCR and showed an increase in carcinoma tissues, indicating its overexpression due to gene amplification.MAL2 is a heterologous binding partner of TPD52 [23]. However, the potential role for MAL2 in TPD52 effects on migration/invasion and the related signaling pathways is still unclear and needs to be further investigated. TPD52 expression in high-grade serous carcinoma was shown to be highand was associated with poor OS in CRC patients. Multivariate analysis showed that TPD52 expression was an independent prognostic factor for reduced OS of CRC patients. However, high-level TPD52 staining was shown to be significantly associated with improved overall survival in patients with stage III serous ovarian carcinoma [60], and TPD52 expression was significantly decreased in the hepatocarcinoma cell carcinoma (HCC) tissues and renal cell carcinoma (RCC) cells and was associated with improved OS and disease-free survival (DFS) in HCC patients [61,62]. Therefore, the differential correlation of high TPD52 expression and OS of patients in several cancers may demonstrate different roles for TPD52 in cancer progression.

In conclusion, our data highlighted MAL2 and TPD52 frequent overexpression in CRC compared with non-cancerous tissues and TPD52 is tightly associated with cell motility. These data suggested that MAL2 and TPD52 might be potential biomarkers for cancer detection and clinical prognosis, and might be promising therapeutic targets for CRC.
